# Treatment of refractory *Helicobacter pylori* infection: A new challenge for clinicians

**DOI:** 10.3389/fmicb.2022.998240

**Published:** 2022-10-18

**Authors:** XinBo Xu, Cong He, Yin Zhu

**Affiliations:** Department of Gastroenterology, Digestive Disease Hospital, The First Affiliated Hospital of Nanchang University, Nanchang, China

**Keywords:** refractory *Helicobacter pylori* infection, rescue therapy, antibiotic resistance, antibiotic susceptibility testing, empirical treatments

## Abstract

Patients who have failed two or more attempts to eradicate *Helicobacter pylori* are commonly referred to as refractory. Although the incidence of refractory *Helicobacter pylori* infection is only 10–20%, with the increasing rate of antibiotic resistance in various regions, the treatment of refractory *Helicobacter pylori* infection has gradually become a difficult problem faced by clinicians. When choosing a rescue therapy, the physician must consider numerous factors. A longer treatment duration, higher doses of proton pump inhibitors (PPIs), or the use of potassium-competitive acid blocker (P-CAB) may increase the efficacy of triple therapy or bismuth quadruple therapy. Rescue treatment based on bismuth quadruple therapy usually achieves better results. At the same time, treatment based on drug susceptibility tests or genotypic resistance is recommended where available. Of course, appropriate empiric treatment can also be selected according to local drug resistance, a patient’s previous medication history and compliance. It is the best choice if it can improve the success rate of the first treatment and reduce the occurrence of refractory *Helicobacter pylori* infection. This review aims to summarize the articles related to refractory *Helicobacter pylori* in recent years and to explore a better remedial treatment plan for clinicians.

## Introduction

Eradication with *Helicobacter pylori* can reduce the recurrence rate of peptic ulcers, reduce the incidence of *Helicobacter pylori*-associated gastritis, cure patients with mucosa-associated lymphoid tissue lymphoma (MALT), and reduce the risk of gastric cancer (Lee et al., 2016; Malfertheiner et al., 2017; Liou et al., 2019). Currently, the eradication rate of clarithromycin triple therapy, a commonly used first-line treatment regimen, is less than 80% (Malfertheiner et al., 2017; Liou et al., 2020), and quadruple therapy with levofloxacin and bismuth agents is often selected as the second-line treatment (Liou et al., 2010; Fallone et al., 2016). However, approximately 10–20% of patients still fail treatment (Liou et al., 2011, 2016). Patients who fail two or more treatments are often referred to as patients with refractory *Helicobacter pylori* infection, and treating these patients is still a difficult problem in the clinic (Losurdo et al., 2022a). Therefore, we reviewed the evidence from previous studies to identify more appropriate treatment options.

### Status of refractory *Helicobacter pylori* infection

*Helicobacter pylori* is a major carcinogen that can cause gastric cancer, with 1–3% of *Helicobacter pylori* patients eventually developing stomach cancer (Blaser, 2016). Therefore, the eradication of *Helicobacter pylori* plays a very important role in the prevention and control of gastric cancer. Current first-line treatment regimens have good eradication rates. However, antibiotic resistance rates have risen around the world. The success rate of initial eradication is challenged by multiple resistant bacteria (Savoldi et al., 2018; Lin et al., 2021). Accordingly, the occurrence of refractory *Helicobacter pylori* infection is increasing, becoming a concern that cannot be ignored (Liou et al., 2011, 2016). Therefore, it is necessary to perform in-depth research on refractory *Helicobacter pylori* infection to explore its causes and potential treatment modalities.

### The causes of treatment failure

#### Antibiotic resistance

Antibiotic resistance is currently the main cause of refractory *Helicobacter pylori* infection, and antibiotic resistance is mainly a concern for clarithromycin, metronidazole and levofloxacin (Sugano et al., 2015; Malfertheiner et al., 2017). A report published in 2018 looked at antibiotic resistance in 65 countries and territories around the world. The primary and secondary drug resistance rates of clarithromycin, metronidazole and levofloxacin in other regions of the world were ≥ 15%, except for the Americas, Southeast Asia and some parts of Europe. However, due to the large heterogeneity of studies in different regions, the results need to be discussed and analyzed separately by region (Kuo et al., 2017; Savoldi et al., 2018; Hulten et al., 2021; Megraud et al., 2021). In the Asia-Pacific region, the drug resistance of clarithromycin, metronidazole and levofloxacin is also serious, and the drug resistance rate of metronidazole is as high as 44% (Table 1). In China, studies have shown that secondary resistance to clarithromycin, metronidazole and levofloxacin is greater than 50% and even greater than 90% in some areas. Not only has the prevalence of single-resistant strains increased, but that of double- and multiple-resistant strains has also increased. This has become an important reason for the annual increase in *Helicobacter pylori* eradication failure. In these highly resistant areas, eradication therapies containing clarithromycin, metronidazole and levofloxacin are clearly no longer suitable (Baylina et al., 2019; Li et al., 2020, 2021; Kuo et al., 2021; Resina and Gisbert, 2021). How to choose antibiotics to eradicate *Helicobacter pylori* is a new challenge for clinicians (Savoldi et al., 2018).

**TABLE 1:** Antibiotics resistance of *Helicobacter pylori.*

References	Region	Antibiotic resistance		
		Clarithromycin	Metronidazole	Levofloxacin
Megraud et al. (2021)	European	21.4%	38.9%	15.8%
Hulten et al. (2021)	The United States	17.4%	43.9%	57.8%
Kuo et al. (2017)	Asia	17%	44%	18%
Camargo et al. (2014)	Latin America	12%	53%	15%
Savoldi et al. (2018)	Africa	15%	91%	14%

#### *Helicobacter pylori*-related factors

*Helicobacter pylori* can increase its resistance to antibiotics through mutation of drug resistance genes. Studies have shown that *Helicobacter pylori* can increase its resistance to metronidazole by upregulating the expression of hefA, a key gene of the drug efflux pump, and mutation of rdxA (Lee et al., 2018a). Mutations in the A2142G and A2143G loci may lead to increased clarithromycin resistance (Hamza et al., 2018). Other studies have also shown that gyrA, 23S rRNA and 16S rRNA mutations in *Helicobacter pylori* are also responsible for other increased resistance (Nezami et al., 2019). In addition, *Helicobacter pylori* can also escape the effects of antibiotics through internalization. Research by Apolinaria Garcia-Cancino revealed that *Helicobacter pylori* can hide in Candida albicans under clarithromycin and amoxicillin and avoid their effects (Sánchez-Alonzo et al., 2021). At the same time, another study revealed that *Helicobacter pylori* entered the gastric mucosal tissues of patients in whom *Helicobacter pylori* eradication had failed, mainly in the gastric body (95.2%). Standard clarithromycin-containing triple therapy failed even though the internalized *Helicobacter pylori* was mostly clarithromycin-sensitive, suggesting that cellular internalization of *Helicobacter pylori* may have contributed to the failure (Beer et al., 2021). In addition, *Helicobacter pylori* will activate the chromosomal type I toxin antitoxin system (AapA1 IsoA1) under the oxidative stress, and further express AapA1 toxin to induce the formation of coccoids, so as to avoid the influence of antibiotics. This process did not destroy the integrity of *Helicobacter pylori* biofilm and did not produce changes in membrane potential, which may be related to the interference of cell elongation/division interference. But the specific mechanism still needs more research and discussion (El Mortaji et al., 2020).

#### Host factors

Host factors are also important causes of refractory *Helicobacter pylori* infection. Most proton pump inhibitors (PPIs) need to be metabolized through the CYP2C19 pathway, and the metabolic type of CYP2C19 can affect the eradication effect of *Helicobacter pylori* by affecting the metabolism of PPIs. Patients with the fast metabolic type need to increase the dose of PPIs to maintain a high eradication rate (Fontes et al., 2019). Studies have shown that vitamin D can affect *Helicobacter pylori* colonization and eradication by affecting the autophagy pathway, and the eradication rate of *Helicobacter pylori* is low in patients with vitamin D deficiency (Hu et al., 2019; Shatla et al., 2021). The family environment is also one of the possible causes of eradication failure. *Helicobacter pylori* is easily transmitted among family members. Studies have shown that there is a significant correlation between a history of eradication failure in parents and eradication failure in offspring (Deguchi et al., 2019; Ding et al., 2022). Therefore, emphasis should be placed on the eradication of *Helicobacter pylori* in the home. Older age, prior eradication treatment, and a history of PPI use also increased the risk of eradication failure (Yan et al., 2020). Otherwise, studies have suggested that smoking, non-alcoholic fatty liver disease, and human immunodeficiency virus (HIV) infection may have contributed to the failure of eradication, but more research is needed to confirm these results (Itskoviz et al., 2017; Hanafy and Seleem, 2019; Takara et al., 2019; Nkuize et al., 2021).

## Options for rescue treatment: Antibiotic-susceptibility testing or empirical treatment

### Antibiotic-susceptibility testing

How should rescue treatment be chosen? We generally have two options: experiential treatment and drug sensitivity-guided regimens, both of which have their own advantages. With the increase in antibiotic resistance rates in various regions of the world, drug sensitivity-guided regimens are increasingly being chosen by more clinicians. The advantage of drug sensitivity guidance is to be able to know the individual’s sensitivity to antibiotics and to use sensitive antibiotics specifically to increase the success rate of eradication. Not only is this approach recommended in the guidelines, but many regional studies provide strong evidence to support this view (Lee et al., 2016; Malfertheiner et al., 2017; Liou et al., 2019). The study revealed that in patients with more than two eradication failures, the eradication rate of both the triple and quadruple regimens guided by drug sensitivity reached more than 90%, especially in patients with penicillin allergy. The treatment guided by drug sensitivity achieved almost perfect results, with an eradication rate as high as 99% (Huang et al., 2018; Yu et al., 2019; Luo et al., 2020; Gingold-Belfer et al., 2021; Lee et al., 2021). However, not all hospitals meet the criteria necessary to carry out drug sensitivity testing because it requires great laboratories and professional testing personnel. This is the reason why drug-sensitive guided treatment is not widely available (Gisbert, 2020).

### Empirical treatment

Empirical treatment is more acceptable because it does not require additional testing to evaluate drug sensitivities. However, clinicians need to predict the effectiveness of treatment options based on local epidemiology, population resistance, and whether patients have been previously exposed to antibiotics for any reason (Gisbert, 2020). Although empirical therapy cannot provide individualized precision treatment compared with drug sensitivity therapy, it is an alternative in areas lack of medical facilities for laboratory testing. Moreover, there are also more studies showing that empirical treatment of refractory *Helicobacter pylori* infection has a good effect, with an eradication rate of 75–90% (Gisbert, 2020; Ji et al., 2020; Nyssen et al., 2021). Since the main resistant antibiotics are clarithromycin, levofloxacin and metronidazole, more studies are needed to investigate the efficacy of other antibiotics as an empirical treatment option.

### Dosage and selection of proton pump inhibitors

Due to polymorphism in the CYP2C19 gene among patients, the dose of PPIs will affect the efficacy of eradication therapy. In clinical trials, 20 mg (low dose) and 40 mg (high dose) are usually used for comparison (Graham et al., 2019). High doses of PPI significantly improved the outcome of standard triple therapy (Katelaris and Katelaris, 2017;Ierardi et al., 2019; Losurdo et al., 2022b). Therefore, a double dose of PPIs is recommended for rescue therapy. In addition, the selection of new-generation PPIs to replace existing drugs is also an option to improve treatment effectiveness. Such as Rabeprazole, Esomperazole, they are less affected by CYP2C19 polymorphisms. A Japanese study showed that a 7-day triple therapy based on vonoprazan proved superior to lansoprazole-based triple therapy for 7 days. This is especially true in patients infected with clarithromycin-resistant strains (Murakami et al., 2016). Another Japanese study showed that the annual eradication rates of second-line therapy between 2013 and 2018 were 90, 82.6, 88.8, 87.5, 91.8, and 90.1%, respectively. The use of vanorazan was an independent factor in the success of second-line treatment (Mori et al., 2019).

### New antibiotics

It is very important to optimize the treatment plan and choose more efficient antibiotics for rescue treatment. We need to find some antibiotics that are more effective as rescue treatment and in how they are administered. In past reports of rescue treatment, tetracycline and rifabutin were assessed in many studies, and both achieved good results. In recent years, many studies have reported the role of tetracycline in remedial therapy. Two studies from Taiwan, China, compared the efficacy of tetracycline regimens in rescue therapy. The first study compared the 10-day TL regimen (tetracycline plus levofloxacin bismuth quadruple regimen) with the AL triple regimen (amoxicillin plus levofloxacin triple regimen) in remedial therapy. The eradication rate in the TL group was 98% higher than that in the AL group (69.2%) (Hsu et al., 2017). Another study compared the 10-day TL regimen with the AL regimen as a remedial treatment for *Helicobacter pylori*. The eradication rate of 89.3% in the TL group was only 69.6% in the AL group, and the eradication rate of levofloxacin-resistant strains in the TL group was also higher than that in the AL group (Hsu et al., 2021). The 10–14-day regimen was associated with a higher eradication rate than the 7-day regimen with tetracycline (Shin et al., 2021). Studies have shown that the minimum inhibitory concentration (MIC) of tetracycline can achieve a better effect as long as it reaches 0.094 mg/L (Hsieh et al., 2020). At the same time, the Korean study compared the eradication rate of the tetracycline regimen with different dosing methods, and the dosage of 2,000 mg tetracycline per day, whether 500 mg qid or 1,000 mg bid, had a good eradication effect (Kim et al., 2022). Otherwise, many studies have reported that the Pylera three-in-one capsule combined with PPI achieved a good curative effect in remedial treatment.

In addition to tetracycline, many studies have reported the role of other antibiotics in rescue treatment (Table 2). A meta-analysis showed that quinolones are the best second-line treatment option in Western countries (Yeo et al., 2019). Antofloxacin is a new quinolone drug. A Chinese study compared antofloxacin with a 14-day triple therapy with levofloxacin. The eradication rate of the levofloxacin group was higher than that of the antofloxacin group (87.6 vs. 68.5%) when the levofloxacin resistance rate was over 40%. Antofloxacin has both good efficacy and safety (Mori et al., 2020; He et al., 2022). Second, previous reports have demonstrated that furazolidone has a good eradication effect in first-line treatment. It also had a significant effect on rescue therapy in patients who had failed previous clarithromycin or levofloxacin quadruple therapy. The 14-day quadruple furazolidone regimen achieved 90% eradication in patients with clarithromycin and levofloxacin resistance rates of more than 40% (Kong et al., 2020; Resina and Gisbert, 2021). However, some countries do not allow the use of furazolidone for safety reasons, so more studies are needed to explore the safety of furazolidone use. Otherwise, a large number of studies have reported the efficacy of rifabutin in rescue therapy (Ierardi et al., 2014; Nyssen et al., 2022; Table 3). Studies have shown that rifabutin triple therapy has an obvious antibacterial effect on multiple drug-resistant *Helicobacter pylori*, and the eradication rate can reach more than 80% (Fiorini et al., 2018; Siavoshi et al., 2018; Ribaldone et al., 2019; Kuo et al., 2020). At present, the main rescue therapy contains rifabutin, amoxicillin and PPIs. More studies are needed to compare the efficacy of other antibiotics combined with rifabutin in rescue therapy. In conclusion, in the absence of drug sensitivity guidance, clinicians should consider the use of the above antibiotics to address refractory *Helicobacter pylori* infection.

**TABLE 2:** Eradication rate of tetracycline-containing rescue therapy.

References	Dosing frequency	Duration of therapy	Eradication rate %
Nyssen et al. (2020)	Tetracycline 500 mg qid; metronidazole 500 mg tid; bismuth 240 bid; PPI 20 mg bid	10/14	Over all: 77%; PP:NA ITT:NA
Kim et al. (2022)	Tetracycline 1 g bid; metronidazole 500 mg tid; bismuth 600 mg bid; PPI 20 mg bid	14	Over all: 92.5% PP:NA ITT:NA
Hsu et al. (2021)	Tetracycline 500 mg qid; levofloxacin 500 mg qd; bismuth 300 mg qid; PPI 40 mg bid	10	Over all:NA ITT:89.3% PP:89.1%
Shin et al. (2021)	Tetracycline 500 mg qid; metronidazole 500 mg tid; bismuth 120 mg qid; PPI 20 mg bid	7/10/14	Over all:NA ITT:80.7% PP:93.3%
Losurdo et al. (2022b)	Pylera (Tetracycline/ metronidazole/bismuth) bid; PPI 20 mg bid	10	Over all:NA ITT:84.9% PP:86.1%
De Francesco et al. (2021)	Pylera (Tetracycline/ metronidazole/bismuth) bid; PPI 20 mg bid	–	Over all:NA ITT:90.6% PP:94%
Fiorini et al. (2017)	Pylera (Tetracycline/ metronidazole/bismuth) bid; PPI 20 mg bid	10	Over all:NA ITT:81% PP:87%

**TABLE 3:** Eradication rate of rifabutin-containing rescue therapy.

References	Dosing frequency	Duration of therapy	Eradication rate %
Kuo et al. (2020)	Rifabutin 150 mg bid; amoxicillin 1 g bid; PPI 40 mg bid	10	Over all:79.5% ITT:NA PP:NA
Fiorini et al. (2018)	Rifabutin 150 mg bid; amoxicillin 1 g bid; PPI 40 mg bid	12	Over all:NA ITT:82.9% PP:88.7%
Hirata et al. (2020)	Rifabutin 150 mg bid; amoxicillin 750 mg bid; PPI 20 mg bid	10	Over all:NA ITT:100% PP:100%
Saracino et al. (2020)	Rifabutin 150 mg bid; amoxicillin 1 g bid; PPI 40 mg bid	12	Over all:NA ITT:51.9% PP:61.9%
Ribaldone et al. (2019)	Rifabutin 150 mg bid; amoxicillin 1 g bid; PPI 20 mg bid	14	Over all:NA ITT:71.5% PP:72.7%

### Duration of therapy

Insufficient time is also an important factor in eradication failure. A treatment duration extended by 14 days with triple therapy was superior to the same regimen of 7 or 10 days with first-line therapy (Yuan et al., 2013). Therefore, various guidelines recommend a duration of 14 days for first-line treatment, unless shorter durations are locally proven to be non-inferior and yield reliably high success rates (Fallone et al., 2016; Liou et al., 2018). Among second- or third-line treatments, the cure rates of levofloxacin triple therapy at 7, 10, and 14 days were 58.3, 68.2, and 93.3%, respectively (Noh et al., 2016). However, the benefit of extending treatment to 14 days was minimal in susceptible strains (Liou et al., 2018). In strains resistant to clarithromycin, the eradication rate can be increased due to the effect of PPI-amoxicillin dual therapy. In summary, we recommend 14 days of treatment for refractory *Helicobacter pylori*.

### Other treatments

Does the addition of adjunctive agents on a triple or quadruple basis increase the efficacy of remedial therapy? In recent years, many studies have combined probiotics, biological extracts, traditional Chinese medicine and other adjuvant drugs with traditional therapy to increase the eradication effect of rescue treatment. A study from China treated patients with refractory *Helicobacter pylori* with Lactobacillus for 2 weeks followed by 10-day quadruplex therapy with tetracycline and furazolidone as rescue treatment. The overall eradication rate was 92% in the intention-to-treat (ITT) analysis and 91.8% in the Per-Protocol (PP) analysis, with fewer adverse reactions and a good safety profile (Liu et al., 2020). The Iranian study also found that in patients in whom eradication had failed, quadruple therapy containing Lactobacillus was more effective as a rescue therapy than non-probiotic treatment (Karbalaei and Keikha, 2021). In addition, there are also studies on the role of traditional Chinese medicine as an adjuvant therapy in remedial therapy. A Chinese meta-analysis revealed that integrated traditional Chinese and Western medicine treatment had a higher eradication rate and fewer adverse reactions than Western medicine alone (OR 2.21, 95% CI: 1.74, 2.81) (Zhong et al., 2022). Other studies demonstrated that the combination of berberine or WUZHUYUTANG combined with the antibiotic bismuth can improve the eradication effect of rescue treatment (Nagata et al., 2018; Zhang et al., 2020). However, these studies were only conducted in China, and more Western studies are needed to confirm whether this treatment is suitable for patients in other parts of the world. It was also found that the extracts of lime could inhibit the growth and urease activity of clarithromycin, metronidazole and levofloxacin triple-drug-resistant strains; therefore, these extracts could have therapeutic potential (Lee et al., 2018b).

## Conclusion

In conclusion, in the remedial treatment of refractory *Helicobacter pylori* infection, it is recommended to use a higher dose of PPI quadruple therapy for 14 days, and vonoprazan is a better choice when necessary. When conditions permit, it is recommended to use drug sensitivity tests or genotype resistance guidance therapy. Of course, taking into account the economy, compliance and feasibility of patients, appropriate empiric treatment can be an acceptable alternative to drug sensitivity treatment based on previous drug use and prevailing drug resistance in the region. Tetracycline, furazolidone, rifambutin, or a new generation of quinolone-based therapy or bismuth quadruple therapy may be a good option. Further large randomized studies are needed to determine the best treatment for refractory *Helicobacter pylori* infection.

## Author contributions

CH: give the idea. XX: write the article. YZ: offer a suggestion. All authors contributed to the article and approved the submitted version.
